# Supplementary material for: Pyruvate Dehydrogenase Kinase 1 inhibition mediated oxidative phosphorylation enhancement in cartilage promotes osteoarthritis progression
Source: BMC Musculoskelet Disord. 2023 Jul 20;24:597. doi: 10.1186/s12891-023-06585-6 (PMC10357736; doi:10.1186/s12891-023-06585-6)
Supplement: Supplementary file 1 — Additional file 1. [file 12891_2023_6585_MOESM1_ESM.zip › MF.Gsea.1653627316242/GOMF_PROTEIN_DISULFIDE_ISOMERASE_ACTIVITY.html]

Details for gene set GOMF\_PROTEIN\_DISULFIDE\_ISOMERASE\_ACTIVITY[GSEA]

|  || Dataset | 55457\_collapsed\_to\_symbols.cls.cls #OA\_versus\_normal.cls.cls #OA\_versus\_normal\_repos |
| Phenotype | cls.cls#OA\_versus\_normal\_repos |
| Upregulated in class | OA |
| GeneSet | GOMF\_PROTEIN\_DISULFIDE\_ISOMERASE\_ACTIVITY |
| Enrichment Score (ES) | 0.6042419 |
| Normalized Enrichment Score (NES) | 1.8530474 |
| Nominal p-value | 0.0023980816 |
| FDR q-value | 0.027863989 |
| FWER p-Value | 0.399 |
Table: GSEA Results Summary

  

Fig 1: Enrichment plot: GOMF\_PROTEIN\_DISULFIDE\_ISOMERASE\_ACTIVITY      
 Profile of the Running ES Score & Positions of GeneSet Members on the Rank Ordered List

  

| SYMBOL | TITLE | RANK IN GENE LIST | RANK METRIC SCORE | RUNNING ES | CORE ENRICHMENT || 1 | CRELD2 | cysteine rich with EGF like domains 2 [Source:HGNC Symbol;Acc:HGNC:28150] | 146 | 0.867 | 0.1437 | Yes |
| 2 | PDIA5 | protein disulfide isomerase family A member 5 [Source:HGNC Symbol;Acc:HGNC:24811] | 324 | 0.735 | 0.2614 | Yes |
| 3 | ENOX1 | ecto-NOX disulfide-thiol exchanger 1 [Source:HGNC Symbol;Acc:HGNC:25474] | 558 | 0.638 | 0.3574 | Yes |
| 4 | ERP29 | endoplasmic reticulum protein 29 [Source:HGNC Symbol;Acc:HGNC:13799] | 706 | 0.592 | 0.4517 | Yes |
| 5 | P4HB | prolyl 4-hydroxylase subunit beta [Source:HGNC Symbol;Acc:HGNC:8548] | 777 | 0.575 | 0.5491 | Yes |
| 6 | ERP44 | endoplasmic reticulum protein 44 [Source:HGNC Symbol;Acc:HGNC:18311] | 1175 | 0.481 | 0.6042 | Yes |
| 7 | PDIA6 | protein disulfide isomerase family A member 6 [Source:HGNC Symbol;Acc:HGNC:30168] | 2073 | 0.353 | 0.5978 | No |
| 8 | PIGK | phosphatidylinositol glycan anchor biosynthesis class K [Source:HGNC Symbol;Acc:HGNC:8965] | 2801 | 0.270 | 0.5896 | No |
| 9 | TXNDC5 | thioredoxin domain containing 5 [Source:HGNC Symbol;Acc:HGNC:21073] | 3378 | 0.214 | 0.5831 | No |
| 10 | PDIA4 | protein disulfide isomerase family A member 4 [Source:HGNC Symbol;Acc:HGNC:30167] | 3756 | 0.181 | 0.5862 | No |
| 11 | CRELD1 | cysteine rich with EGF like domains 1 [Source:HGNC Symbol;Acc:HGNC:14630] | 5555 | 0.028 | 0.4517 | No |
| 12 | GLRX2 | glutaredoxin 2 [Source:HGNC Symbol;Acc:HGNC:16065] | 6116 | -0.014 | 0.4107 | No |
| 13 | QSOX1 | quiescin sulfhydryl oxidase 1 [Source:HGNC Symbol;Acc:HGNC:9756] | 6130 | -0.015 | 0.4124 | No |
| 14 | PDIA3 | protein disulfide isomerase family A member 3 [Source:HGNC Symbol;Acc:HGNC:4606] | 6607 | -0.050 | 0.3844 | No |
| 15 | PDIA2 | protein disulfide isomerase family A member 2 [Source:HGNC Symbol;Acc:HGNC:14180] | 8961 | -0.229 | 0.2427 | No |
| 16 | ITGB3 | integrin subunit beta 3 [Source:HGNC Symbol;Acc:HGNC:6156] | 10310 | -0.352 | 0.2010 | No |
Table: GSEA details [plain text format]

  

Fig 2: GOMF\_PROTEIN\_DISULFIDE\_ISOMERASE\_ACTIVITY      
 Blue-Pink O' Gram in the Space of the Analyzed GeneSet

  

Fig 3: GOMF\_PROTEIN\_DISULFIDE\_ISOMERASE\_ACTIVITY: Random ES distribution      
 Gene set null distribution of ES for **GOMF\_PROTEIN\_DISULFIDE\_ISOMERASE\_ACTIVITY**
